# Wnt and Kras signaling-dark siblings in lung cancer

**DOI:** 10.18632/oncotarget.305

**Published:** 2011-07-13

**Authors:** Eugenia C. Pacheco-Pinedo, Edward E. Morrisey

**Affiliations:** ^1^Department of Medicine, Department of Cell and Developmental Biology, Institute for Regenerative Medicine, Cardiovascular Institute, University of Pennsylvania, Philadelphia, PA 19104

**Keywords:** Kras, Wnt/β-catenin, lung progenitor, Clara cell

## Abstract

Aberrant Kras signaling is observed in a high percentage of human lung cancers while activating mutations in the Wnt/β-catenin signaling pathway are only rarely found. Our recent work has shown that the combined activation of both Kras and Wnt/β-catenin signaling leads to a dramatic increase in both tumor incidence and size. Moreover, lung tumors generated by the combined activation of both of these pathways exhibit a distinct phenotype similar to embryonic progenitors found in the developing lung. Thus, combinatorial activation of Kras and Wnt/β-catenin pathways leads to a significant increase in lung tumor formation characterized by a more progenitor like phenotype.

The “multiple hit” hypothesis of cancer pathogenesis has long been favorably viewed as a reasonable explanation of the relative rarity of many cancers. This hypothesis is derived from multiple lines of thought. For instance, two carcinogens, applied in sequence, are often more successful at initiating cancer in experimental animals than application of a single carcinogen during the same time [1, 2]. Another line of evidence comes from epidemiological observations of a linear correlation between the rate of cancer death and the patients age [3, 4]. Lung cancer is one of the leading causes of cancer death in the Western world. Although the majority of lung cancers are associated with cigarette smoking, only a minority of smokers end up having lung cancer. This suggests that there are other genetic or environmental causes that drive the high incidence of lung cancer. A recent report from our laboratory suggests that the “multiple hit” model of cancer is also useful in explaining the combined effects of co-activation of β-catenin and an oncogenic K-ras on lung tumorigenesis.

While Wnt/β-catenin pathway has been causally linked to colon and skin cancer in the past, its role in the development of lung cancer has been less clear [5, 6]. There is significant interest in exploring the role of Wnt signaling in cancer and many drug discovery efforts are underway to block the pathway in multiple types of cancers [7]. Previous reports have shown that Wnt/β-catenin activation in postnatal bronchiolar lung epithelium is ineffective in inducing lung tumor formation [8]. However, studies have linked Wnt/β-catenin activation to increased lung tumors metastasis and proliferation [9]. Despite these and other findings, little is understood about both the ability of activated Wnt/β-catenin signaling to promote lung tumorigenesis and which cell lineages within the complex milieu of the lung epithelia are susceptible to oncogenic transformation by Wnt signaling.

Taking advantage of cell lineage specific cre lines for the secretory epithelium of the upper airways (CC10-cre, [10]), we recently showed that while activation of β-catenin alone using the *Ctnnb1*^Δ(ex3)^ mice which will delete important regulatory phosphorylation sites within β-catenin that inhibit its function in Wnt signaling upon cre mediated recombination, has little effect on lung tumor formation, co-activation of both oncogenic K-ras and activated β-catenin leads to far more aggressive tumor formation than when either pathway is activated alone [11]. Until recently, the available cre lines that could be used in the postnatal lung were not specific for any of the various epithelial lineages that populate the lung airways in a distinct proximal-distal manner. However, Li et. al. recently showed that when the cre recombinase is knocked into the Clara Cell 10 (CC10) kilodalton protein gene, cre recombinase can be expressed postnataly in a Clara or secetory cell specific fashion in the lung [10]. This line is similar to that reported by Rawlins et. al. which used a tamoxifen inducible version of cre instead of a constitutive version [12]. The advantage of these new cre lines is that investigators can address in a cell specific fashion the consequences of oncogene activation in the postnatal lung, specifically the secretory epithelium.

Using the constitutive CC10-cre line, our group showed that β-catenin activation had little effect on secretory epithelial proliferation or tumor formation [10, 11]. This is in agreement with previous reports and suggests that Wnt/β-catenin activation in the secretory epithelium of the lung is not normally an oncogenic event. To determine whether activation of another oncogenic pathway would result in tumor development originating in the secretory epithelium, we next expressed the G12D oncogenic form of Kras (KrasG12D) using the CC10-cre line. K-ras mutations have been described in approximately 21% of lung cancers and are thus one of the most common neoplastic mutations found in lung cancers [13, 14]. Previous studies from Kim et. al. have indicated the presence of a bronchioalveolar stem cell (BASC) as a potential cancer stem cell in the lung [15]. Expression of KrasG12D in these cells leads to formation of focal hyperplasitic regions and adenomas [16, 17]. However, none of these studies asked specifically whether such tumor initiation originated in the secretory epithelium. In our studies, expression of KrasG12D lead to distinct areas of epithelial hyperplasia with some of these regions forming adenomas by 3-4 months of age. However, we did not observe the presence of lung adenocarcinomas in these mice.

**Figure 1:**
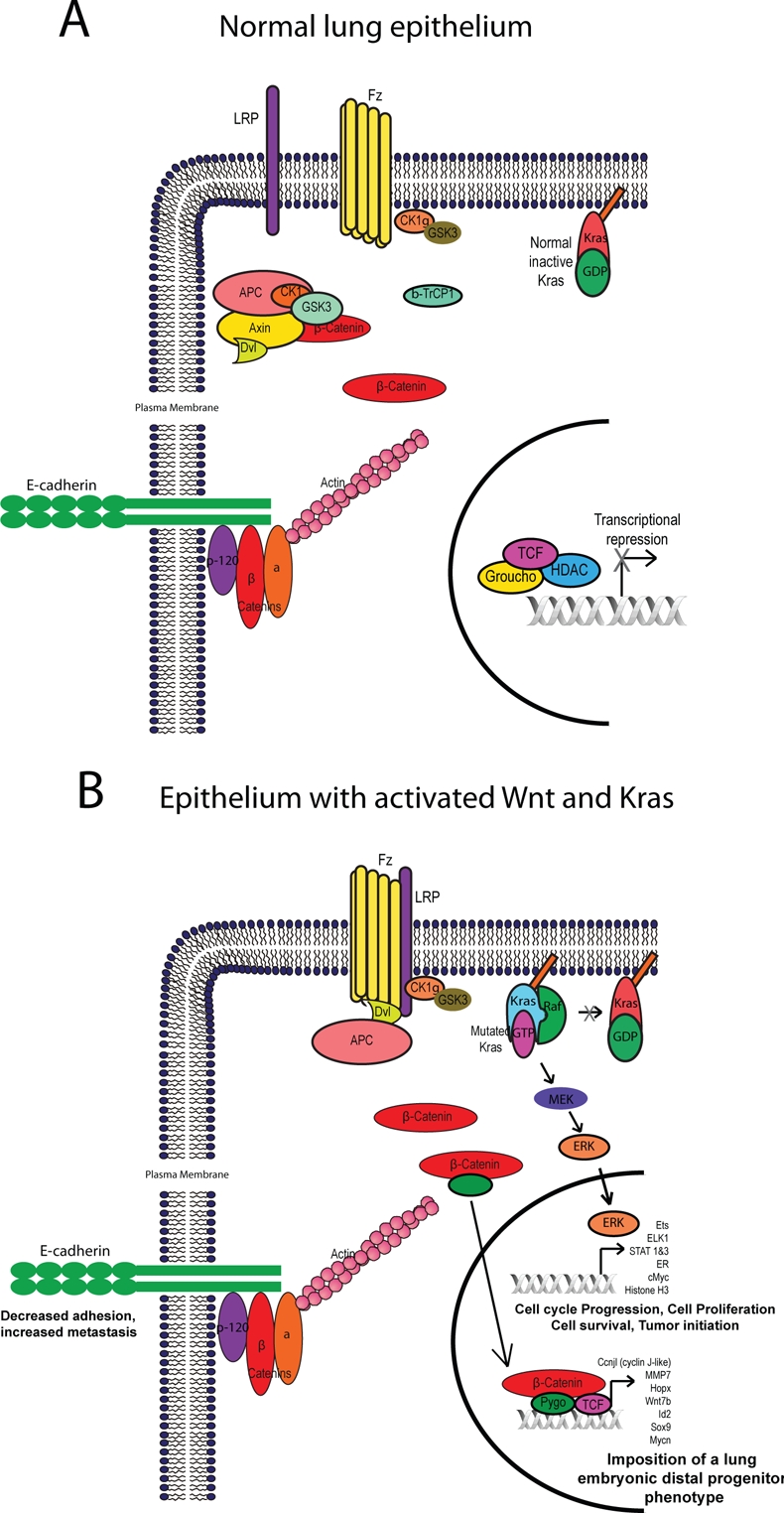
Model of airway epithelial phenotypic trans-differentiation events in KrasG12D:*Ctnnb1^Δ(ex3)^* double mutant lung tumors Normal lung epithelium consists of both secretory cell lineages in the bronchiolar regions (Clara cells) as well as ciliated epithelium while the alveolar regions consist of both type 1 and type 2 pneumocytes (A). Expression of KrasG12D leads to expansion of secretory cells as well as BASC cells (B). Expression of both KrasG12D:*Ctnnb1^Δ(ex3)^* leads to a trans-differentiation event where secretory cells alter their phenotype to resemble distal endoderm progenitors found in the embryonic lung (C).

Lung tumors associated with Kras activation, both in humans and mice, show a great deal of phenotypic variation. This suggests the presence of other factors that can inhibit or promote Kras induced tumorigenesis in the lung. Therefore, we generated mice that expressed both KrasG12D and the activated β-catenin allele *Ctnnb1*^Δ(ex3)^. In these compound mutant animals, lung tumors developed rapidly and often progressed to the adenocarcinoma stage. These tumors were larger and, interestingly, had decreased expression of secretory epithelial markers such as CC10 and increased expression of alveolar epithelial markers such as surfactant protein C (Sftpc). Despite these changes in gene expression, fate-mapping of the CC10-cre positive cells in this tumor model showed that the mutant cells did arise from CC10+ secretory epithelium.

Using gene profiling techniques, we demonstrated that the *KrasG12D:Ctnnb1*^Δ(ex3)^ mutant tumors were distinctly different than either the KrasG12D or *Ctnnb1*^Δ(ex3)^ mutants alone. One of the most interesting aspects of these tumors is their high level of similarity to distal embryonic lung endoderm progenitors. These embryonic endoderm cells express genes such as Sox9 and Id2 which are extinguished in normal adult lung epithelium. Remarkably, the *KrasG12D:Ctnnb1^Δ(ex3)^* double mutant tumors expressed high levels of these markers. Moreover, *KrasG12D:Ctnnb1^Δ(ex3)^* double mutant tumors displayed decreased expression of Hopx, a transcription factor normally expressed at high levels in adult lung epithelium that has been suggested to be a tumor suppressor [18]. Thus, activation of both oncogenic K-ras and Wnt/β-catenin pathways leads to a trans-differentiation event in adult secretory epithelium, altering the phenotype to resemble distal embryonic lung endoderm during the early stages of lung development.

The idea that expression of an embryonic progenitor phenotype leads to a more aggressive form of lung cancer is supported by a recent report showing that a lung embryonic progenitor expression profile predicts a poor prognosis in lung adenocarcinoma [19]. The authors of this study used differential gene expression analysis between E11.5 and E17.5 distal lung endoderm progenitors and derived a list of 10 genes which are enriched at E11.5. Patients with decreased levels of these genes had a better survival rate that patients with increased expression of these genes. Although the genes we identified are not found in this list, the basic concept that a tumor that is more similar to early embryonic lung progenitors would be more aggressive and lead to worse survival is supported by these findings.

**Figure 2:**
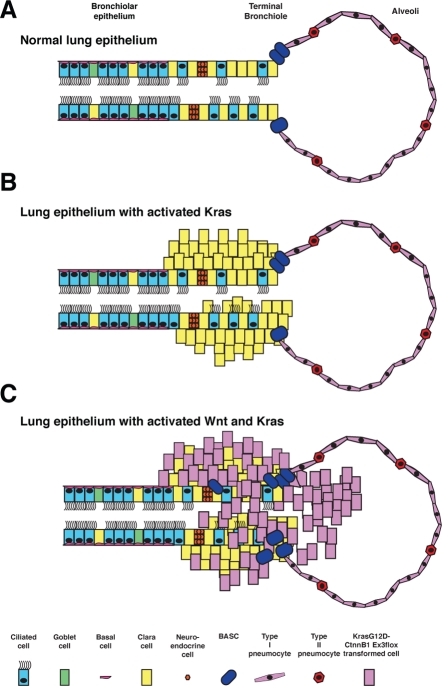
Activation state of Wnt and K-ras pathways in airway epithelium of the lung In normal airway epithelial secretory cells, the Wnt pathway is not activated and K-ras signaling is not constitutively active (A). Expression of both KrasG12D and *Ctnnb1^Δ(ex3)^* activated alleles leads to expression of host of target genes which cooperate to trans-differentiate adult secretory epithelium to an embryonic distal endoderm progenitor phenotype (B). The resulting alterations in both pathways leads to decreased E-cadherin expression which may be due to sequestering of β-catenin away form the cell-cell junctions to the Wnt pathway and decreasing stability of the cadherin-catenin complex.

These changes are interesting and present new information regarding the role of Wnt/β-catenin in lung cancer. However, the finding that certain genes representing the distal embryonic lung endoderm gene expression profile including Sox9 were also upregulated in several human lung cancers including lung adenocarcinomas further extends the idea of an increased embryonic gene expression profile that could be a common hallmark of human lung cancer.

Several lines of evidence indicate that Wnt/β-catenin signaling is crucial for distal lung endoderm development in the lung. Loss of Wnt2/2b or β-catenin prior to lung endoderm specification leads to complete lung agenesis including loss of tracheal development and loss of induction of Nkx2.1 expression [20]. Slightly later in development, loss of β-catenin or expression of the Wnt inhibitor Dkk1, leads to decreased distal lung endoderm development and expanded proximal airway endoderm development [21, 22]. These data point to an essential role for Wnt/β-catenin in specification and/or maintenance of the distal lung endoderm. This concept is in line with what is observed in the *KrasG12D:Ctnnb1^Δ(ex3)^* double mutant lung tumors where the combined expression of oncogenic K-ras and β-catenin causes a trans-differentiation of proximal secretory epithelium to an embryonic distal lung endoderm phenotype. Thus, in the presence of activated Wnt/β-catenin signaling, the default phenotype in the lung epithelium may be that displayed by embryonic distal lung endoderm progenitors even if the oncogenic event occurs in proximal secretory epithelium.

Our results suggest that activation of β-catenin signaling in the presence of another oncogenic mutation such as KrasG12D, recapitulates some of the developmental effects of Wnt/β-catenin signaling. The ability of β-catenin and KrasG12D to alter the bronchiolar epithelial phenotype to a distal embryonic lung endoderm phenotype supports the idea that Wnt/β-catenin signaling is necessary and sufficient to drive distal lung endoderm development. Although previous studies have not reported increased tumor formation by ectopic activation of β-catenin signaling in the postnatal lung, several reports have shown that embryonic activation of Wnt/β-catenin signaling can lead to lung epithelial dysplasia as well as ectopic reprogramming of stomach endoderm to the lung lineage. Expression of activated β-catenin starting at E14.5 in the fetal lung caused pulmonary adenomatous tumors in a subset of mice, however, the incidence of tumors was low, indicating that other epigenetic or genetic changes must occur to promote tumor initiation and/or progression [22]. Mutant lungs expressing the *Ctnnb1^Δ(ex3)^* allele after lung specification using the Nkx2.1-cre line also showed formation of polyp like structures indicating a precancerous lesion [23]. Thus, the timing of Wnt/β-catenin activation likely plays an important role as to whether tumors or precancerous lesions will form in the lung.

There have been a few reports showing correlation between increased lung tumor formation and mutations or changes in expression of Wnt signaling components. Dkk3, an inhibitor of the Wnt/β-catenin pathway which is highly expressed in the normal lung has been found down-regulated in lung tumors [24], while the ligand Wnt1 was determined to be a prognostic factor for patients with non small cell lung cancers, NSCLC [25]. Interestingly, human bronchiolar epithelial cells exposed to cigarette smoke have a significant increase in Wnt signaling activation [26]. This would suggest that cigarette smoking alone could prime lung epithelial cells for another oncogenic hit, possibly Kras mutations, that lead to lung cancer.

Lung cancer has been the leading cause of cancer deaths in the United States for many years [27, 28]. Despite ongoing research and improved clinical techniques for early detection, the one-year all-stage survival rate has only increased from 32% in 1973 to 41% in 1994 while the five-year survival has remained unchanged at 14%. We believe that the present findings could lead to screening for Wnt/β-catenin signaling activity as a prognostic indicator for the aggressiveness of lung tumors. Using screening assays for the activation state of the Wnt pathway in lung tumors could help to predict which tumors will develop into a more aggressive phenotype allowing for different treatments. Moreover, with the recent findings that Wnt/β-catenin signaling are associated with increased lung tumor metastasis [9], such screening could also inform decisions on additional diagnostic tests and use of biopsies to detect metastatic spread of the tumors.

Given the enormous clinical burden that lung cancer represents, new advances are needed in both diagnosis and treatment. The finding that two of the most common oncogenic pathways, Wnt/β-catenin and Kras, can cooperate to promote aggressive tumor development in mice suggests that these pathways should be analyzed together in lung tumor diagnosis. Combinatorial approaches using therapies directed towards the Wnt/β-catenin pathway as well as the Kras pathways may also prove useful in combating lung cancer. Recent data suggests that rapamycin may be a useful treatment in certain cancers with aberrant Wnt/β-catnein signaling activity [29-31]. This and other such approaches may provide new therapeutic ideas for lung cancer treatment in the future.
